# Socio-economic and demographic factors associated with reproductive and child health preventive care in Mozambique: a cross-sectional study

**DOI:** 10.1186/s12939-020-01303-3

**Published:** 2020-11-09

**Authors:** Chanvo Daca, Miguel San Sebastian, Carlos Arnaldo, Barbara Schumann

**Affiliations:** 1 Ministry of Health, Directorate of Planning and Cooperation, Maputo, Mozambique; 2 Department of Epidemiology and Global Health, Umea University, Umea, Sweden; 3 Universidade Eduardo Mondlane, Maputo, Mozambique

**Keywords:** Socio-economic, Immunization, Insecticide treated nets, Modern contraceptives, Mozambique

## Abstract

**Background:**

Reproductive and child health interventions are essential to improving population health in Africa. In Mozambique, although some progress on reproductive and child health has been made, knowledge of social inequalities in health and health care is lacking.

**Objective:**

To investigate socio-economic and demographic inequalities in reproductive and child preventive health care as a way to monitor progress towards universal health coverage.

**Methods:**

A cross-sectional study was conducted, using data collected from the 2015 Immunization, AIDS and Malaria Indicators Survey (IMASIDA) in Mozambique. The sample included 6946 women aged 15 to 49 years. Outcomes variables were the use of insecticide treated nets (ITN) for children under 5 years, full child immunization and modern contraception use, while independent variables included age, marital status, place of residence, region, education, occupation, and household wealth index. Prevalence ratios (PR) with 95% confidence intervals (95% CI) were calculated by log binomial regression to assess the relationship between the socio-economic and demographic characteristics and the three outcomes of interest.

**Results:**

The percentage of mothers with at least one child under 5 years that did not use ITN was 51.01, 46.25% of women had children aged 1 to 4 years who were not fully immunized, and 74.28% of women were not using modern contraceptives. Non-educated mothers (PR = 1.33; 95% CI: 1.16–1.51) and those living in the Southern region (PR = 1.36; 95% CI: 1.17–1.59) had higher risk of not using ITN, while the poorest quintile (PR = 1.34; 95% CI: 1.04–1.71) was more likely to have children who were not fully immunized. Similarly, non-educated women (PR = 1.17; 95% CI: 1.10–1.25), non-working women (PR = 1.09; 95% CI: 1.04–1.16), and those in the poorest quintile (PR = 1.13; 95% CI: 1.04–1.24) had a higher risk of not using modern contraceptives.

**Conclusion:**

Our study showed a low rate of ITN utilization, immunization coverage of children, and modern contraceptive use among women of reproductive age. Several socio-economic and demographics factors (region, education, occupation, and wealth) were associated with these preventive measures. We recommend an equity-oriented resource allocation across regions, knowledge dissemination on the importance of ITN and contraceptives use, and an expansion of immunization services to reach socio-economically disadvantaged families in order to achieve universal health coverage in Mozambique.

## Introduction

In Africa, tremendous efforts have been carried out to improve child and reproductive health outcomes [1, 2]. According to the World Health Organization (WHO), Africa has managed to reduce the maternal mortality ratio from 990 to 510 per 1.00.000 live births between 1990 and 2013, and mortality under the age of five has seen a 57% reduction, from 182 to 85 per 1000 live births between 1990 and 2018 [3, 4].

However, despite these achievements, socio-economic and demographic health inequalities persist in the region. In a multi-country study conducted by the WHO in 2010, infant mortality in Madagascar was 71% higher for children born to mothers with no formal education compared to mothers with at least some secondary education. In Nigeria, the wealthiest households were 4.4 times more likely to receive diphtheria pertussis tetanus (DPT3) vaccines when compared to the poorest quintile. In Tanzania, women living in urban areas were twice as likely to use a modern contraceptive method as those living in rural areas [5].

In Mozambique, although some progress has been made over the last decade, child and reproductive health indicators underperform. For instance, the maternal mortality ratio ranges from 249 to 480 per 100,000 live births [3]. The proportion of children receiving measles and DPT-3 vaccination increased from 60 to 82% between 1997 and 2015, but few districts have achieved the target of 80% complete vaccination coverage. Although the proportion of children who slept under a mosquito net rose from 47.9% in 2015 to 73% in 2018, malaria cases rose from 3.5 million in 2000 to 6 million in 2014 [6, 7]. Lastly, the contraceptive prevalence rate improved significantly from 11.3 to 25.3% between 2011 and 2015 [8, 9], but it is still much lower than the average of 59.3% in southern African countries in 2015 [10].

In the country of Mozambique, research on socio-economic and demographic inequalities in reproductive and child health is still limited. In a study assessing the relationship between socio-economic status and child mortality in 2003, manual work and lack of education were associated with a higher child mortality [11], whereas another study revealed how childhood malnutrition was concentrated among the poor [12].

In the last decade, the country has undertaken several initiatives aiming to improve health and decrease socio-economic inequalities in health. For example, in 2007, Mozambique joined the International Health Partnership, now called Universal Health Coverage 2030 Partnership (UHC) [13]. The UHC Partnership supported the development of a national health policy to guide UHC and primary health care interventions (see Appendix), with a particular focus on strengthening the health financing system [14]. The latest Health Sector Strategic Plan for 2014 to 2019, which emphasized a reform and decentralization agenda, with particular attention paid to women and young people, specifically targeted a reduction in socio-economic and demographic health disparities [15].

Nevertheless, despite a clear commitment to pursuing UHC goals, Mozambique is facing critical challenges because of a high burden of disease, a weak health system, a shortage of human resources, deficient management of medical equipment, lack of laboratory consumables and medicines, as well as wide disparities in health outcomes and access to health care within and between provinces [8].

Monitoring and evaluating a country’s progress towards UHC is fundamental to improving health policy decisions and to promoting an equitable health system [14]. Moreover, evaluations must incorporate all social groups, since national averages can mask inequalities in the most vulnerable population.

The objective of this study was to investigate social inequalities in reproductive and child preventive health care in Mozambique. Specifically, we aimed to analyse socio-economic, demographic and geographic differences in child bed net use, child immunization and modern contraceptive use among women in 2015.

## Material and methods

### Study setting

**Fig. 1:**
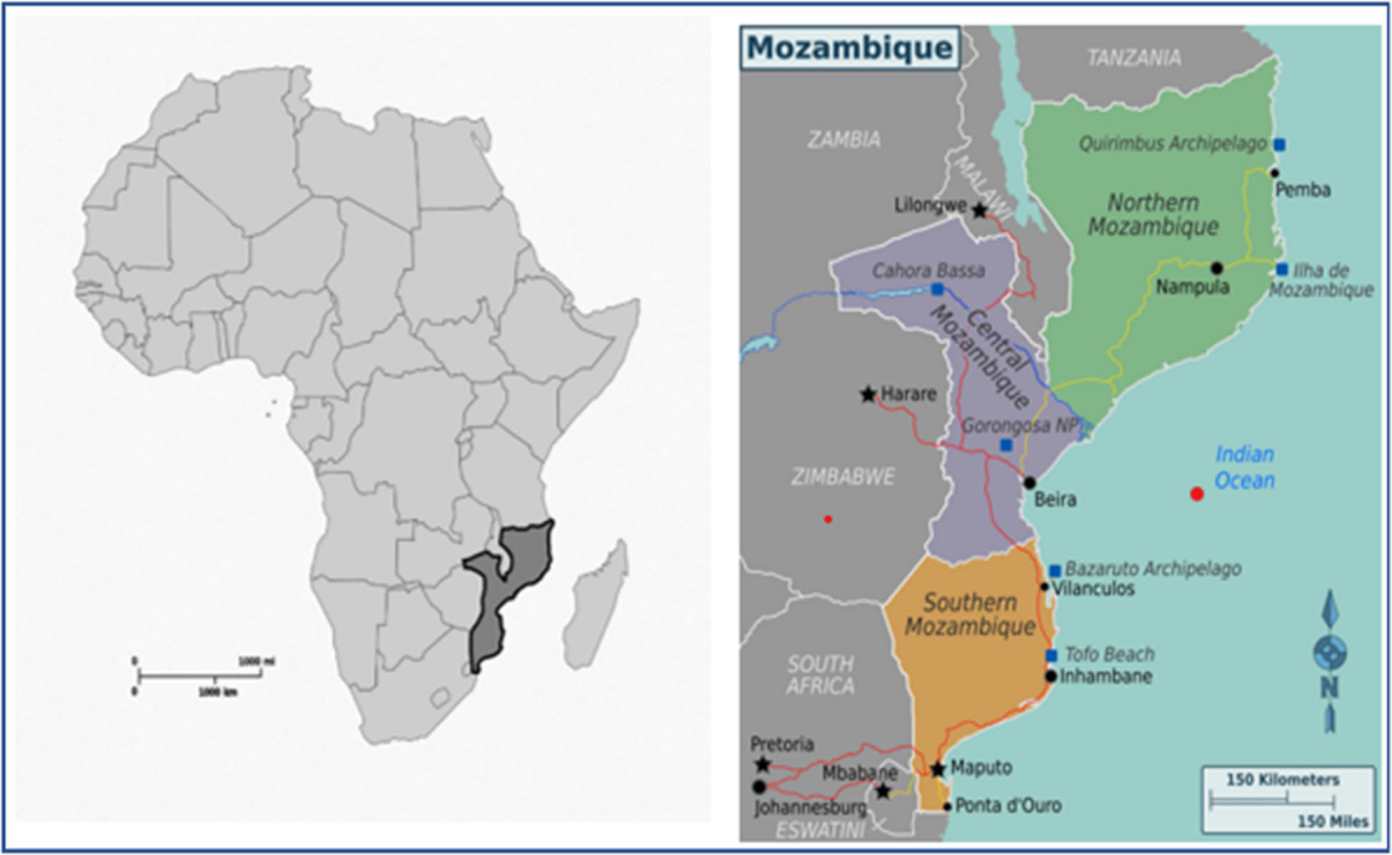
Map of Mozambique showing the three regions (source: www.wikimedia.org)

Mozambique, located in south eastern Africa, is administratively divided into 10 provinces and 154 districts. Provinces are divided into three geographical regions: Northern, Central and Southern (Fig. 1). Due to historical reasons, socio-economic resources have been unequally distributed among regions, of which the Southern (where the capital is) had benefited more. The population of Mozambique in 2017 was close to 29 million and women comprised about 51% of the total population. About 70% of the population live and work in rural areas [16].

The Mozambican health service delivery system (The National Health Service) is dominated by the public sector consisting of four levels. Level I has very basic resources and is staffed by clinical officers, nurses and medical technicians, allocated in health posts and health centers at community level; level II includes district hospitals that offer basic diagnostic, surgical, and obstetric care; level III consists of provincial hospitals, which offer specialized curative care, high level of diagnostics services and serve as training centers; finally, level IV consists of four referral hospitals in the big cities of Maputo, Beira, Nampula and Quelimane. However, access to health care, particularly in rural areas, is complicated due to deficiencies in provided services, long distances and inadequate road transport.

Insecticide treated bed nets, child vaccinations and contraceptives are provided free of charge mainly at levels I and II of the health system. For some curative services as well as for all drug prescriptions, user fees are charged, which are subsidized by the government.

### Study population

This cross-sectional study utilized data from the IMASIDA survey of 2015, part of the Demographic and Health Survey Program (DHS) conducted from June to September 2015. IMASIDA was a household survey of adult men and women aged 15 to 59, who were permanent residents in the country. A three-stage multistage cluster sampling design was used to provide representative national and provincial-level estimates, as well as stratification for rural and urban areas within provinces. Detailed methodological procedures of the survey have previously been described [9].

In the IMASIDA, a national representative sample of 7368 households was selected. From the households, 8204 women aged 15 to 59 were eligible, and 7749 agreed to be interviewed (94.4% response rate). IMASIDA surveys collect primary data using biomarker samples and three types of questionnaires: household, women’s and men’s questionnaires. The women’s questionnaire was the only that provided relevant data for our specific research objectives. The present study consisted of a total of 6949 women at reproductive age (15 to 49 years old), all of whom were included in the analytical sample for contraceptive use. Due to the operationalization of the outcomes, the analytical sample for bed net utilization was 4709 and for immunization, it was 2694.

The IMASIDA data are publicly available and were downloaded with permission from the Demographic and Health Survey at www.dhsprogram.com/data/available-datasets.cfm. The IMASIDA data were collected through face-to-face interviews using three questionnaires: the household’s, the women’s, and the men’s. For the purposes of this study, only the women’s questionnaire was used. This questionnaire collected data on age, place of residence, marital status, occupation, education, household assets, vaccination of children, family’s bed net use, antenatal care, birth history, use of contraceptive methods, recent sexual activity, and fertility preferences. Portuguese was the language used in the interviews, and all survey instruments were pre-tested in urban and rural areas.

### Outcome variables

Three different outcomes were used in this study capturing the lack of access to health preventive interventions, namely bed net use, full child immunization and modern contraceptive use.

Insecticide treated net use (ITN) was categorized as “no” if all children under five had slept under a bed net the day before the survey, and as “yes” if at least one child had not slept under a bed net. A child aged 12 to 59 months was considered fully immunized if it had received all the recommended doses and vaccines according to the national immunization schedule: Bacille Calmette-Guérin (BCG) (birth dose), three doses of DPT, three doses of polio vaccine, and one dose of measles vaccine [17]. If any of the recommended doses could not be verified by a vaccination card or reported by the mother, then the child was classified as “not fully immunized”. Only the last child of each woman was considered in this study.

Lack of modern contraceptive use was captured by asking the woman if she had used any contraceptive methods at last intercourse. Modern contraceptives included female and male sterilization, contraceptive pills, intrauterine contraceptive device, injectables (Depo-Provera), implants (Norplant) and condoms. Periodic abstinence (rhythm, calendar method), withdrawal (coitus interruptus) and folk methods were classified as non-modern methods. If a respondent reported using both a modern and a non-modern method, this was counted as a modern method use. The responses were recoded into a binary variable (yes/no). Modern contraceptives will be referred to as contraceptives hereafter.

### Socio-economic and demographic variables

The age of the mother was categorized in three groups: 15 to 24, 25 to 39, and 40 to 49 years old. Marital status was divided into three categories: single/never in union, married (married/living with partner), and other (widowed, divorced, no longer living together). Place of residence was split into rural or urban residence. Participants belonged to ten provinces which were organized in the three official geographical regions: Northern, Central and Southern.

Education was classified in three categories: no education, completed primary school, and completed secondary school or above. Nine categories of occupation were captured in the IMASIDA, but due to the low sample size in some categories, four groups were created: (a) non manual (managerial, clerical, sales, and services); (b) farmers; (c) manual (household and domestic, skilled and unskilled manual), and (d) not working. Wealth index, a proxy for income, was calculated from the household questionnaire based on the following assets in the participant’s household: television and car; dwelling characteristics such as flooring material; type of drinking water source, and toilet facilities. The wealth index was obtained by principal component analysis and stratified into quintiles, from richest to poorest [18].

### Statistical analysis

Descriptive statistics were calculated for the socio-demographic variables and the three outcomes. Prevalence Ratios (PR) with 95% confidence interval (CI) I were calculated by log binomial regression to assess the relationship between the socio-economic characteristics and the three outcomes of interest [19]. Statistically significant variables in the crude model were included in the adjusted one.

Sample weighting was applied in all analyses in order to adjust for the unequal probability of sample selection and interview. A detailed explanation of the weighting procedure can be found in the DHS Guide [18]. All analyses were performed with Stata version 15.

Collinearity among variables was tested, and all variance inflation factor were below 3, which is under the recommended threshold of 10 [19].

### Ethical clearance

Data for this study were obtained from the IMASIDA 2015, available at the DHS website (http://www.measuredhs.com). No ethical approval was required for this specific study.

## Results

In Table 1, the prevalence of the socio-economic and demographic characteristics and health outcomes of the participants are presented.

**Table 1:** Weighted prevalences of socio-economic and demographic characteristics by lack of use of health preventive care

	No ITN use N (%)	No Full Immunization N (%)	No Contraceptives Use N (%)
**Age group**			
15–24	2053 (43.18)	1016 (36.38)	2874 (41.57)
25–39	2091 (43.98)	1454 (52.06)	2838 (41.04)
40–49	611 (12.84)	323 (11.57)	1202 (17.39)
**Marital Status**			
Single	687 (14.44)	168 (6.03)	1178 (17.04)
Married	3295 (69.30)	2106 (75.41)	4565 (66.02)
Other	773 (16.26)	518 (18.56)	1171 (16.94)
**Residence**			
Urban	1506 (31.66)	795 (28.46)	2437 (35.24)
Rural	3249 (68.34)	1998 (71.54)	4478 (64.76)
**Region**			
Northern	1670 (35.13)	1044 (37.39)	2442 (35.31)
Central	1829 (38.47)	1069 (38.31)	2502 (36.18)
Southern	1255 (26.40)	679 (24.31)	1971 (28.51)
**Education**			
Secondary	983 (20.68)	503 (18.01)	1576 (22.79)
Primary	2523 (53.05)	1514 (54.23)	3544 (51.25)
No education	1249 (26.27)	775 (27.76)	1795 (25.96)
**Occupational Class**^a^			
Non manual	788 (16.58)	502 (17.98)	1248 (18.08)
Farmers	1080 (22.74)	669 (23.98)	1476 (21.38)
Manual	269 (5.68)	172 (6.18)	399 (5.78)
Not working	2612 (55.00)	1447 (51.87)	3782 (54.77)
**Wealth Quintile**			
Richest	560 (11.78)	259 (9.28)	975 (14.09)
Richer	672 (14.13)	375 (13.44)	1063 (15.38)
Middle	896 (18.84)	525 (18.81)	1275 (18.44)
Poorer	1272 (26.76)	758 (27.15)	1696 (24.52)
Poorest	1355 (28.49)	875 (31.33)	1906 (27.56)
**Total**	**4755**	**2792**	**6915**

^a^Missing occupational values

The proportion of women aged 15 to 24 years was 41.57 and 17.39% were in the oldest group (40 to 49). Almost two thirds of the women (64.76%) lived in rural areas. About 22.96% of women had no formal education, 51.25% had primary education and 22.79% had completed secondary school. Over half (54.77%) of the participants reported not working.

Regarding the outcomes, around half (51.01%) of women had at least one child under five who did not sleep under the ITN, 46.25% had children aged 1 to 4 who were not fully immunized, and 74.28% of the women were not using a modern contraceptive method.

All socio-economic and demographics factors were associated with lack of insecticide treated net use in the bivariate analysis. However, in the multivariable analysis, only the Southern region (PR = 1.36; 95% CI: 1.17–1.59) and no education (PR = 1.33; 95% CI: 1.16–1.51) remained associated with the lack of ITN use (Table 2).

**Table 2:** Women’s socio-economic and demographic characteristics and their association with the non-use of health preventive care. Crude (PR) and adjusted prevalence ratios (Adj. PR) with 95% confidence intervals

	No ITN use		No Full Immunization		No Contraceptive Use	
Age group	PR (95% CI)	Adj. PR (95% CI)	PR (95% CI)	Adj. PR (95% CI)	PR (95% CI)	Adj. PR (95% CI)
15–24	1.00	1.00	1.00	–	1.00	1.00
25–39	1.01 (0.94–1.08)	0.96 (0.90–1.04)	1.02 (0.92–1.13)	–	**0.90 (0.87–0.94)**	**0.89 (0.86–0.93)**
40–49	**1.15 (1.04–1.26)**	1.07 (0.97–1.18)	1.06 (0.91–1.22)	–	1.04 (0.99–1.08)	0.98 (0.94–1.02)
Residence						
Urban	1.00	1.00	1.00	1.00	1.00	1.00
Rural	**1.25 (1.11–1.41)**	1.14 (0.94–1.38)	**1.32 (1.15–1.52)**	1.05 (0.88–1.26)	**1.19 (1.13–1.25)**	1.00 (0.94–1.05)
Regions						
Northern	1.00	1.00	1.00	1.00	1.00	1.00
Central	**1.26 (1.09–1.45)**	**1.26 (1.09–1.45)**	1.10 (0.95–1.28)	1.08 (0.94–1.24)	1.05 (0.99–1.11)	**1.06 (1.01–1.12)**
Southern	**1.22 (1.05–1.42)**	**1.36 (1.17–1.59)**	**0.67 (0.57–0.80)**	**0.81 (0.68–0.97)**	**0.77 (0.72–0.82)**	**0.87 (0.83–0.92)**
Education						
Secondary	1.00	1.00	1.00	1.00	1.00	1.00
Primary	**1.23 (1.11–1.36)**	**1.18 (1.06–1.30)**	1.08 (0.93–1.24)	0.87 (0.74–1.01)	**1.25 (1.19–1.31)**	**1.13 (1.07–1.19)**
No education	**1.42 (1.27–1.60)**	**1.33 (1.16–1.51)**	**1.45 (1.26–1.68)**	1.05 (0.87–1.27)	**1.36 (1.28–1.44)**	**1.17 (1.10–1.25)**
Marital Status						
Single	1.00	1.00	1.00	–	1.00	
Married	0.94 (0.86–1.03)	**0.86 (0.79–0.95)**	0.99 (0.83–1.18)	**–**	1.03 (0.98–1.08)	
Other	**1.12 (1.00–1.25)**	1.03 (0.92–1.16)	1.20 (0.99–1.45)	–	1.02 (0.96–1.08)	
Occup. Class						
Non-manual	1.00	1.00	1.00	1.00	1.00	1.00
Farmers	**1.19 (1.06–1.35)**	1.07 (0.94–1.22)	**1.25 (1.06–1.48)**	1.08 (0.90–1.29)	**1.23 (1.16–1.31)**	1.05 (0.99–1.11)
Manual	0.97 (0.82–1.14)	0.93 (0.79–1.10)	1.03 (0.82–1.3)	1.003 (0.79–1.26)	0.98 (0.88–1.09)	0.99 (0.91–1.09)
Not working	1.07 (0.96–1.18)	1.06 (0.96–1.17)	0.99 (0.86–1.13)	0.93 (0.81–1.08)	**1.18 (1.12–1.25)**	**1.09 (1.04–1.16)**
Wealth Quintile						
Richest	1.00	1.00	1.00	1.00	1.00	1.00
Richer	1.03 (0.87–1.23)	1.02 (0.86–1.22)	0.95 (0.76–1.18)	0.92 (0.74–1.15)	1.06 (0.97–1.15)	0.99 (0.91–1.08)
Middle	**1.17 (1.07–1.49)**	1.12 (0.91–1.38)	0.98 (0.79–1.20)	0.91 (0.72–1.16)	**1.21 (1.12–1.31)**	**1.09 (1.00–1.19)**
Poorer	**1.24 (1.06–1.44)**	1.09 (0.86–1.38)	**1.31 (1.08–1.58)**	1.10 (0.86–1.40)	**1.39 (1.29–1.50)**	**1.13 (1.03–1.24)**
Poorest	**1.25 (1.07–1.45)**	1.12 (0.89–1.41)	**1.63 (1.36–1.95)**	**1.34 (1.04–1.71)**	**1.41 (1.31–1.52)**	**1.13 (1.03–1.24)**

Non full child immunization was associated in the crude model with several socio-economic, demographic and geographical factors, such as place of residence, region, level of education, occupation class, and wealth. When adjusted for the significant variables, living in the Southern region (PR = 0.81; 95% CI: 0.68–0.97) was a protective factor of child immunization, while belonging to the poorest quintile (PR = 1.34; 95% CI: 1.04–1.71) increased the risk of not being fully immunized. Finally, all predictor variables were associated in the bivariate model with lack of contraceptives use, except marital status. After adjustment, women living in the Southern region were more likely to use contraceptives than those in the Northern region (PR = 0.87; 95% CI: 0.83–0.92). Furthermore, non-educated women (PR = 1.17; 95% CI 1.10–1.25), non-working women (PR = 1.09, 95% CI: 1.04–1.16), and those from the poor (PR = 1.13; 95% CI: 1.03–1.24) and poorest quintiles (PR = 1.13; 95% CI: 1.03.24) had a higher risk of not using contraceptives compared to the reference group.

## Discussion

Our study, based on the Mozambican IMASIDA survey 2015, showed a low prevalence of bed net utilization by children (51.01%), low immunization coverage (46.25%) and low rates of modern contraceptive use (25.75%). Several socio-economic and demographic factors were associated with the outcomes. Living in the Southern region and a low education level were associated with non-bed net use. Similarly, women living in the Southern region were more likely to fully vaccinate their child, whereas the poorest quintile had a higher risk of not fully vaccinating their child. Finally, the non-educated, poorest and not working women had higher risk of not using contraceptives, whereas women living in the Southern region were more likely to use contraceptives.

### Insecticide treated nets (ITN)

In Mozambique, malaria was reported as the leading cause of death in children under 5 years old in 2011 [20]. The use of ITN, together with a household sprayed with insecticide, continues to be one of the essential national vector control strategies. In this study, 51.0% of mothers had at least one child who did not sleep under an ITN, which is below the Roll Back Malaria target of having at least 60% of pregnant women and children under five using nets treated with insecticide [6, 21].

Two main factors were associated with the use of bed nets: region and education. The lower utilization of ITN in the Southern region could be explained because the malaria national control program has been prioritizing ITN distribution to regions with higher malaria burden, which is not the case for the Southern region [22, 23].

Among the socio-economic and demographics factors, only education showed a significant association with ITN use. This has been shown in another study carried out in the Central region, where ITN use was strongly associated with a high education level [23]. Similarly, studies from Africa have shown that a higher education level was associated with ITN, for instance in Congo [23], Gambia [24], and Cameroon [25].

In Mozambique, ITN, as one of the malaria control strategies, are freely distributed at health posts to target groups, such as pregnant women and children under five during medical appointments and antenatal care visits [26]. Regular health care visits and health literacy tend to be more common in educated women, possibly explaining the high ITN use in this specific group.

### Vaccination coverage

The prevention of child mortality through immunization is one of the most cost-effective public health interventions [27]. Our results showed that 46.25% of mothers had not fully immunized their last child which is a much lower coverage than the 94% goal stated in the national health strategic plan 2014 to 2019 [15].

In Mozambique, full vaccination coverage among children aged 12 to 23 months was reported to be 64% in 2011 [28], lagging behind other African countries, such as Namibia (71.5%) or Rwanda (94.8%) [29]. Even though immunization coverage has been increasing in Mozambique, a range of different barriers still persist. To be vaccinated in Mozambique, the child’s mother has to attend a health centre and since 60% of the population lives more than 8 km away from a health facility, transport issues or work load during the harvesting season may impede health care access [9, 29–32].

Another study assessing the risk factors for incomplete vaccination in the rural southern part of the country found that transportation cost to the health post was a constraint for child vaccination [32]. Also, in Ethiopia, an association between wealth and health has been found, where rich families were more likely to fully vaccinate their children than poor families [33].

This study showed that living in the Southern region was strongly associated with full child vaccination. Regional inequalities in access to health care have been previously highlighted in Mozambique [34–36]. The Southern region, where the capital is located, has been historically privileged in terms of investment, favouring its economic development and subsequently a greater availability of social services, such as health care and schools [36, 37].

### Contraceptive use

The contraceptive prevalence rate in Mozambique has improved significantly, from 11.3% in 2011 to 25.3% in 2015 [38]. In this study we found that only 26% of women were using contraceptives, which is much lower than other countries in sub-Saharan Africa (SSA), such as in Namibia (46%), or than the SSA regional average of 59.3% in 2015 [10, 39].

In this study, the non-educated, poorest and non-working women were less likely to use modern contraceptives. It is widely known that higher education contributes positively to awareness of preventive health measures, and utilization of services, such as contraceptive use, towards better outcomes on overall reproductive and maternal health [39]. The association between education, wealth and contraceptive use has been also revealed in many other African countries. A systematic review undertaken in six African countries revealed that wealthier households were more likely to use contraceptives [40]. Apart from education and wealth, a number of others factors have been reported to influence contraceptive use, such as frequent church attendance and religion [41]. A male dominant role in the society and insufficient oral contraceptives supplies at the health centre level have been associated with a low use of contraceptives in Africa [40, 42]. Although we did not study these factors, they might be also of relevance in the Mozambican setting.

### Methodological considerations

Several limitations should be considered when interpreting the results. First, this is a cross-sectional study, therefore causal effects could not be measured. Second, given that the study was population-based where participants were asked numerous past events, recall bias could have been present. Third, social desirability bias could also be operating since some responses could have been provided only to please the interviewer. The extent of these biases in the findings of the study was not possible to establish. The large, nationally representative sample and the procedures followed by the DHS standard surveys to ensure internal and external validity are, however, important strengths of this study. While we acknowledge that other potential confounders could be operating in this study, our selection was however restricted due to the availability of information in the survey.

## Conclusion

This study has showed a low bed net utilization by children, low immunization coverage and low rates of modern contraceptive use. Several socio-economic and demographics factors, such as region of residence, low levels of education and wealth, and lack of employment were associated with the three outcomes of our study. Effective equity-oriented resource allocation strategies across different regions in the country should be implemented. Also, efforts should be concentrated on home visits for health campaigns to disseminate knowledge on the importance of ITN use and contraceptive use, as well as on the expansion of immunization services, particularly to vulnerable families, if progress towards universal health coverage is to be achieved.

## Data Availability

Requests for the data can be made to the DHS Program at https://dhsprogram.com/what-we-do/survey/survey-display-467.cfm

## Appendix -Areas of UHC to monitor progress on health service coverage, Reproductive, maternal, new-born and child health

**Table:**

	Tracer Area	Tracer indicator	Strategic Plan 2014–19
	a. Family planning	Demand satisfied with modern method among women 15–49 who are married or in a union (%)	% of new users in modern family planning methods, number and % of Health facilities offering at least 3 modern contraceptive methods, % of the required budget for purchasing contraceptive that was covered by state budget
	b. Full child immunization	One-year-old children who have received 3 doses of a vaccine containing diphtheria, tetanus and pertussis (%)	% of children fully vaccinated, Number of districts with the operational outreach team
	c. Malaria prevention	The population at risk sleeping under insecticide-treated bed nets (%)	% of the population at risk potentially reached by Insecticide-treated Nets

Source: WHO 2016, Mozambican Strategic Plan 2014–2019

## References

[1] Kinney MV, Kerber KJ, Black RE, Cohen B, Nkrumah F, Coovadia H, Nampala PM, Lawn JE (2010). Science in action: saving the lives of Africa's mothers N, children working g, et al: sub-Saharan Africa's mothers, newborns, and children: where and why do they die?. PLoS Med.

[2] Bhutta ZA, Black RE (2013). Global maternal, newborn, and child health—so near and yet so far. N Engl J Med.

[3] World Health Organization (2014). Trends in maternal mortality: 1990 to 2013: estimates by WHO, UNICEF, UNFPA, The World Bank and the United Nations Population Division.

[4] World Health Organization (2019). Estimates Developed by the UN Inter-Agency-Group for Child Mortality Estimation. Report.

[5] World Health Organization (2010). Health inequities in the African Region of the World Health Organization: magnitudes, trends and sources.

[6] World Health Organization (2019). World malaria report 2019.

[7] Moonasar D, Maharaj R, Kunene S, Candrinho B, Saute F, Ntshalintshali N, Morris N (2016). Towards malaria elimination in the MOSASWA (Mozambique, South Africa and Swaziland) region. Malar.J..

[8] DFID (2017). Improving sexual, reproductive, maternal, newborn, Child and Adolescent Health In Mozambique.

[9] ICF Macro MoHM NHIM (2016). National Statistics Institute Mozambique AIDS Indicator Survey 2015. (Calverton, Maryland, USA: ICF Macro)(Mozambique).

[10] Tsui AO, Brown W, Li Q (2017). Contraceptive practice in sub-Saharan Africa. Popul Dev Rev.

[11] Macassa G, Ghilagaber G, Bernhardt E, Diderichsen F, Burström B (2003). Inequalities in child mortality in Mozambique: differentials by parental socio-economic position. Soc Sci Med.

[12] Salvucci V (2016). Determinants and trends of socioeconomic inequality in child malnutrition: the case of Mozambique, 1996–2011. J Intern Dev.

[13] Ministerio da Saude (2008). Compacto de Mocambique sobre o Compromisso de intencoes.

[14] World Health Organization (2017). Tracking Universal Health Coverage.

[15] Ministry of Health (2014). Health Sector Strategic Plan 2014–2019.

[16] INE (2017). Censo 2017 Divulgação dos Resultados Preliminares. Mozambique.

[17] World Health Organization (2009). *State of the World's Vaccines and Immunization.* World Health Organization.

[18] Croft TNAMJM, Courtney K (2018). Allen, et al. guide to DHS statistics.

[19] Hair, J., et al., Black. 1995.“Multivariate Data Analysis With Regarding.”. Multivariate Data Analysis.

[20] Martinez BAF, Leotti VB, Nunes LN, Machado G, Corbellini LG (2017). Odds ratio or prevalence ratio? An overview of reported statistical methods and appropriateness of interpretations in cross-sectional studies with dichotomous outcomes in veterinary medicine. Front Veterin Sci.

[21] Ranson H, N’guessan R, Lines J, Moiroux N, Nkuni Z, Corbel V (2011). Pyrethroid resistance in African anopheline mosquitoes: what are the implications for malaria control?. Trends Parasitol.

[22] Baume CA, Marin MC (2008). Gains in awareness, ownership and use of insecticide-treated nets in Nigeria, Senegal, Uganda and Zambia. Malar J.

[23] Brentlinger PE, Correia MAC, Chinhacata FS, Gimbel-Sherr KH, Stubbs B, Mercer MA (2007). Lessons learned from bednet distribution in Central Mozambique. Health Policy Plan.

[24] Ndjinga JK, Minakawa N (2010). The importance of education to increase the use of bed nets in villages outside of Kinshasa, Democratic Republic of the Congo. Malar J.

[25] Tchinda VHM, Socpa A, Keundo AA, Zeukeng F, Seumen CT, Leke RGF, Moyou RS. Factors associated to bed net use in Cameroon: a retrospective study in Mfou health district in the Centre region. Pan Afri Med J. 2012;12:3. PMC3489395 23133712

[26] Sharp BL, Kleinschmidt I, Streat E, Maharaj R, Barnes KI, Durrheim DN, Ridl FC, Morris N, Seocharan I, Kunene S (2007). Seven years of regional malaria control collaboration—Mozambique, South Africa, and Swaziland. Am J Trop Med Hyg.

[27] World Health Organization (2016). State of inequality: childhood immunization.

[28] UNICEF (2014). Situation analysis of children in Mozambique.

[29] Restrepo-Méndez MC, Barros AJ, Wong KL, Johnson HL, Pariyo G, França GV, Wehrmeister FC, Victora CG (2016). Inequalities in full immunization coverage: trends in low-and middle-income countries. Bull World Health Organ.

[30] Ministry of Health: Expanded Programme on Immunization. 2009–2013.

[31] Llop-Girones A, Julia M, Chicumbe S, Dula J, Odallah AAP, Alvarez F, Zahinos I, Mazive E, Benach J (2019). Inequalities in the access to and quality of healthcare in Mozambique: evidence from the household budget survey. Int J Qual Health Care.

[32] Jani JV, De Schacht C, Jani IV, Bjune G (2008). Risk factors for incomplete vaccination and missed opportunity for immunization in rural Mozambique. BMC Public Health.

[33] Lakew Y, Bekele A, Biadgilign S (2015). Factors influencing full immunization coverage among 12–23 months of age children in Ethiopia: evidence from the national demographic and health survey in 2011. BMC Public Health.

[34] Dos Anjos LA, Cabral P (2016). Geographic accessibility to primary healthcare centers in Mozambique. Int J Equity Health.

[35] Macassa G, Ghilagaber G, Bernhardt E, Burstrom B (2006). Inequalities in under-five mortality in Mozambique: differentials by region of residence and ethnic affiliation of the mother. East Afr Med J.

[36] Bujones AK (2013). Mozambique in transition and the future role of the UN*.* Center on international Cooperation.

[37] O'Laughlin B (2010). Questions of Health and Inequality in Mozambique. Instituto de Estudos Sociais e Económicos Cadernos IESE No. 4. Maputo.

[38] Health Mo (2018). Moçambique (IMASIDA) 2015 - Relatório final. Mozambique.

[39] Creanga AA, Gillespie D, Karklins S, Tsui AO (2011). Low use of contraception among poor women in Africa: an equity issue. Bull World Health Organ.

[40] Stephenson R, Baschieri A, Clements S, Hennink M, Madise N (2007). Contextual influences on modern contraceptive use in sub-Saharan Africa. Am J Public Health.

[41] Agadjanian V (2013). Religious denomination, religious involvement, and modern contraceptive use in southern Mozambique. Stud. Fam. Plann.

[42] Chavane L, Dgedge M, Bailey P, Loquiha O, Aerts M, Temmerman M (2017). Assessing women's satisfaction with family planning services in Mozambique. J Fam Plann Reprod Health Care.

